# Prevalence of Enamel Defects in Premolars Whose Predecessors Were Treated with Extractions or Antibiotic Paste

**DOI:** 10.3290/j.ohpd.a45083

**Published:** 2020-09-04

**Authors:** Heloísa Clara Santos Sousa, Marina de Deus Moura de Lima, Cacilda Castelo Branco Lima, Marcoeli Silva de Moura, Ana Victória Lopes Bandeira, Lúcia de Fátima Almeida de Deus Moura

**Affiliations:** a Master, Department of Pathology and Dentistry Clinic, School of Dentistry, Universidade Federal do Piauí, Teresina, Brazil. Performed the experimental design, experiments on partial fulfilment of requirements for a degree, wrote the manuscript.; b Adjunct Professor, Department of Pathology and Dentistry Clinic, Universidade Federal do Piauí, Teresina, Brazil. Proofread the manuscript and contributed substantially to discussion.; c Adjunct Professor, Department of Pathology and Dentistry Clinic, Universidade Federal do Piauí, Teresina, Brazil. Proofread the manuscript and performed all statistical analysis.; d Titular Professor, Department of Pathology and Dentistry Clinic, Universidade Federal do Piauí, Teresina, Brazil. Proofread the manuscript and contributed substantially to discussion.; e Master’s Student, Department of Pathology and Dentistry Clinic, School of Dentistry, Universidade Federal do Piauí, Teresina, Brazil. Performed the experimental design.; f Titular Professor, Department of Pathology and Dentistry Clinic, School of Dentistry, Universidade Federal do Piauí, Teresina, Brazil. Proofread the manuscript and contributed substantially to discussion.

**Keywords:** abnormalities, antibiotic paste, bicuspid, dental enamel, dental pulp necrosis, root canal therapy

## Abstract

**Purpose:**

To determine the prevalence of developmental defects of the enamel (DDE) in premolars whose infected predecessors were submitted to pulp therapy with antibiotic paste or extractions due to pulp necrosis.

**Materials and Methods:**

A cross-sectional study with a consecutive sample consisting of children and adolescents who presented with fully erupted premolars, was evaluated. Data were collected by dental examinations, in which the modified DDE index was applied. Dental records were evaluated and three groups of premolars were determined according to the clinical history of predecessors: GCTZ: with pulp necrosis and treated with CTZ (chloramphenicol, tetracycline, zinc oxide and eugenol) paste; GE: with pulp necrosis and treated by extraction; GH: healthy and physiologically exfoliated. Descriptive analysis and a logistic regression (p <0.05) were performed.

**Results:**

The study included 1017 premolars, DDE was present in 22.5%. Premolars belonging to the GE group presented higher odds of DDE (odds ratio (OR) = 3.52, 95% CI:2.29–5.40) than those of GCTZ group (OR = 2.43, 95% CI:1.51–3.91) and GH group (p <0.01). Enamel defects were more frequent in maxillary premolars (OR = 3.22, 95% CI:1.65–6.27, OR = 3.39, 95% CI:1.67–6.90, OR = 2.90, 95% CI:1.48–5.66 and OR = 3.10, 95% CI:1.54–6.23).

**Conclusions:**

The prevalence of enamel defects was higher in premolars whose predecessors were removed because of necrosis, followed by those treated with CTZ paste and those that were healthy by the time exfoliation occurred.

The furcation region of primary molars have reduced dentine thickness, areas of resorption and is permeated by accessory channels.^15,16^ These characteristics increase the permeability in this region, a condition that favours the development of periradicular lesions involving tissues surrounding developing premolars due to the potential for diffusion of the pulp degradation products and drugs used in pulp therapy of primary molars.^9,13,29^

Developmental defects of the enamel (DDE) are irreversible damage resulting from physicochemical and biological aggressions to ameloblasts of teeth in formation.^21,24^ Such defects present with a multifactorial aetiology,^28^ are risk factors for the installation of biofilm-dependent diseases and frequent causes of aesthetic complaints.^10,27^

CTZ is among the pastes used in pulp therapy of primary molars with irreversible pulpitis or pulp necrosis, which is composed of chloramphenicol, tetracycline, zinc oxide and eugenol. CTZ paste presents with a good antimicrobial action,^3^ biocompatibility^17^ and good clinical and radiographic results.^6,11^ However, the presence of tetracycline may represent a risk factor for the onset of tooth discoloration and/or enamel hypoplasia in the successor premolar due to a high affinity for calcified tissues.^17,23^ The deleterious effects of materials used in the pulp therapy of primary molars on successor premolars has not been extensively studied.^22^

The aim of this study was to determine and to compare the prevalence of DDE of premolars whose predecessors were submitted to pulp therapy with CTZ paste, extractions due to pulp necrosis or they were naturally exfoliated.

## Materials and Methods

### Study Design and Eligibility Criteria

This is a cross-sectional study whose consecutive sample consisted of children and adolescents attending the dental clinic of the Federal University of Piauí (UFPI), who had one or more fully erupted premolars in the period from March to November 2016. To be eligible the patient must be a previous patient of the clinic and their dental records were correctly and signed by the responsible clinician. Otherwise, the child or adolescent would not be examined. Individuals in fixed orthodontic treatment, with imperfect amelogenesis or a moderate or severe degree of dental fluorosis were not included.

### Sample Size Calculation

For the calculation of the minimum sample size of premolars, a formula for comparison of groups according to the qualitative variables in unpaired samples was used. A 95% confidence interval, normal curve point for error (a) 1.96 (5%) and error (b) 0.84 (20%) was considered in the calculation. An average percentage of 31.4% of enamel defects in premolars whose predecessors were submitted to pulp therapy^5,18^ and 50% for those extracted primary molars due to pulp necrosis and without pulp therapy were adopted. A minimum sample of 105 premolars per group was obtained.

### Data Collection

The examiner was calibrated by a researcher with experience in DDE studies. The first phase of training consisted of slide projections with images of the various types of DDE. Upon identifying more than 80% of diagnoses, the examiner progressed to the clinical training phase. Five children and adolescents were evaluated by both examiners and these data were discussed to clarify the clinical diagnostic criteria. Ten subjects were independently examined and re-examined after 2 weeks. Interexaminer (0.82) and intraexaminer (0.90) concordances were determined by the kappa index. A pilot study was conducted with children and adolescents who did not participate in the final sample to evaluate the methods proposed. No adjustments were necessary.

Dental examinations were performed in a conventional dental office under direct reflector illumination, with clean teeth and dried with air jets. The examiner used a flat mouth mirror (Golgran, São Paulo, SP, Brazil) and exploratory probe number 5 (Golgran, São Paulo, SP, Brazil). In the dental examination, the modified DDE index was used,^12^ which classifies enamel development defects in diffuse opacities, demarcated opacities, hypoplasia, other types of defects and their combinations. The extension of the defect was also recorded by the division of the affected dental surface into thirds and the location of the defect in the affected surface. Diffuse opacities, which are characteristic of dental fluorosis, were not considered.

After dental examinations, an analysis of dental records was performed and three groups of premolars were determined according to the clinical history of the predecessors: group 1 (GCTZ): with pulp necrosis and treated with CTZ paste; group 2 (GE): extracted due to pulp necrosis and without pulp therapy; and group 3 (GH): healthy and physiologically exfoliated, without previous restorative treatment or pulp therapy.

Premolars in which pulp necrosis was treated when the child was seven years or less were included in groups GCTZ or GE, a period in which the crown of the premolars was in maturation phase and consequently more vulnerable to DDE.^8,23^

### Ethical Considerations

The study protocol was approved by the Ethics Research Committee of UFPI (protocol No.1.431.204) and conducted in accordance with the Declaration of Helsinki. Written consent was obtained from participants and their parents/guardians.

### Statistical Analysis

Data were analysed using the Statistical Package for the Social Science program (SPSS, Windows, V. 20.0; IBM, Armonk, NY, USA), in which descriptive analysis and logistic regression were performed. Variables with p values ≤ 0.20 in the bivariate analysis were tested in the multivariate analysis and statistically significant variables were maintained in the final model. The odds ratio (OR) and 95% confidence interval were calculated. The level of statistical significance was set at p < 0.05.

## Results

Of the 206 children and adolescents selected for the study, three were not included because they presented dental fluorosis at a moderate degree and one was not included due to the use of fixed orthodontic appliance. The study included 202 patients, with a predominance of males (55.4%), with a family income of less than two minimum wages (55.4%) and a mean age of 10.4 ± 2.3 years. A total of 1017 premolars were examined, of which 107 (10.5%) belonged to GCTZ, 113 (11.1%) to GE and 797 (78.4%) to GH (Table 1). Table 2 presents the proportion of premolars with DDE in the three groups, distributed by type, location and extension of the defect. The presence of DDE was observed in 22.5% of premolars (n = 229), of which demarcated opacities (16.3%), incisal location (34.5%) and an extension of less than 1/3 of the face (78.2%) were the most frequent defects. The DDE frequencies observed for GCTZ, GE and GH were 29.0%, 43.4% and 18.7%, respectively.

**Table 1:** Distribution of DDE frequencies in premolars, according to the groups evaluated and the age of children and adolescents at the treatment date (n = 1017)

VARIABLE	DDE												
		GCTZ				GE				GH			
	Teeth	Yes		No		Yes		No		Yes		No	
	n	n	(%)	n	(%)	n	(%)	n	(%)	n	(%)	n	(%)
	TEETH												
**14**	149	4	(2.7)	6	(4.0)	13	(8.7)	8	(5.4)	30	(20.1)	88	(59.1)
**15**	103	6	(5.8)	1	(1.0)	7	(6.8)	2	(1.9)	20	(19.4)	67	(65.0)
**24**	152	2	(1.3)	5	(3.3)	14	(9.2)	7	(4.6)	28	(18.4)	96	(63.2)
**25**	114	6	(5.3)	5	(4.4)	6	(5.3)	4	(3.5)	22	(19.3)	71	(62.3)
**Maxillary premolars**	518	18	(3.5)	17	(3.3)	40	(7.7)	21	(4.0)	100	(19.3)	322	(62.2)
**34**	145	5	(3.4)	12	(8.3)	2	(1.4)	11	(7.6)	11	(7.6)	104	(71.7)
**35**	106	2	(1.9)	18	(1.0)	1	(0.9)	8	(7.5)	10	(9.4)	67	(63.2)
**44**	143	3	(2.1)	13	(9.1)	4	(2.8)	12	(8.4)	18	(12.6)	93	(65.0)
**45**	105	3	(2.9)	16	(15.2)	2	(1.9)	12	(11.4)	10	(9.5)	62	(59.0)
**Mandibular premolars**	499	13	(2.6)	59	(11.8)	9	(1.8)	43	(8.6)	49	(9.8)	326	(65.3)
**Total**	1017	31	(3.0)	76	(7.5)	49	(4.8)	64	(6.3)	149	(14.7)	648	(63.7)
AGE OF THE CHILD AT THE TIME OF TREATMENT (YEARS)													
**Uninformed**	5	0	(0.0)	0	(0.0)	2	(40.0)	3	(60.0)	–	–	–	–
**≤4**	22	6	(27.3)	12	(54.5)	0	(0.0)	4	(18.2)	–	–	–	–
**5**	39	5	(12.8)	20	(51.3)	9	(23.1)	5	(12.8)	–	–	–	–
**6**	55	8	(14.5)	24	(43.6)	10	(18.2)	13	(23.6)	–	–	–	–
**7**	99	12	(12.1)	20	(20.2)	28	(28.3)	39	(39.4)	–	–	–	–
**Total**	220^a^	31	(14.1)	76	(34.5)	49	(22.3)	64	(29.1)	–	–	–	–

^a^ Total ‘n’ different from 1017. Teeth that had not undergone treatment (GH) were excluded.

**Table 2:** Proportion of premolars with DDE in groups according to the type, the extent and the location of the defects (n = 229)

GROUP	Teeth examined n	DDE									
		Absent		Demarcated opacity		Hypoplasia			Demarcated opacity and Hypoplasia		
		n (%)		n (%)		n (%)			n (%)		
GCTZ	107	76	(71.0)	19	(17.8)	6	(5.6)		6	(5.6)	
GE	113	64	(56.6)	40	(35.4)	7	(6.2)		2	(1.8)	
GH	797	648	(81.3)	107	(13.4)	32	(4.0)		10	(1.3)	
Total	1017	788	(77.5)	166	(16.3)	45	(4.4)		18	(1.8)	
				Less than 1/3 n (%)		At least 1/3 a 2/3 n (%)			At least 2/3 n (%)		
GCTZ	31	–	–	22	(71.0)	8	(25.8)		1	(3.2)	
GE	49	–	–	39	(79.6)	5	(10.2)		5	(10.2)	
GH	149	–	–	118	(79.2)	14	(9.4)		17	(11.4)	
Total	229a	–	–	179	(78.2)	27	(11.8)		23	(10.0)	
				Gingival one-half n (%)		Incisal one-half n (%)		Occlusal n (%)		Cuspal n (%)	
GCTZ	31	–	–	11	(35.5)	14	(45.2)	1	(3.2)	5	(16.1)
GE	49	–	–	14	(28.6)	18	(36.7)	10	(20.4)	7	(14.3)
GH	149	–	–	44	(29.5)	47	(31.5)	24	(16.1)	34	(22.8)
Total	229^a^			69	(30.1)	79	(34.5)	35	(15.3)	46	(20.1)

^a^ Total ‘n’ value different from 1017, since teeth that did not present DDE were excluded.

Premolars belonging to the GE group presented with higher odds of DDE (OR = 3.52; IC95%: 2.29–5.40) than those of the GCTZ (OR = 2.43; IC95%: 1.51–3.91) and GH groups, respectively (p <0.01). Maxillary premolars were more likely to show a DDE (OR = 3.22; IC95%: 1.65–6.27; OR = 3.39; IC95%: 1.67–6.90; OR = 2.90; IC95%: 1.48–5.66 e OR = 3.10; IC95%: 1.54–6.23) than mandibular premolars (p <0.01; Table 3).

**Table 3:** Logistic regression of the independent variables and presence of DDE (n = 1017)

VARIABLE	DDE					
	Yes n (%)	No n (%)	Unadjusted OR (IC95%)	p-value	Adjusted OR (IC95%)	p-value
**GROUP** **GCTZ**	31 (29.0%)	76 (71.0%)	**1.86 (1.18–2.91)**	**<0.01**	**2.43 (1.51–3.91)**	**<0.01**
**GE**	49 (43.4%)	64 (56.6%)	**3.33 (2.20–5.03)**	**<0.01**	**3.52 (2.29–5.40)**	**<0.01**
**GH**	149 (18.7%)	648 (81.3%)	**1**		**1**	
TOOTH						
14	47 (31.5%)	102 (68.5%)	**2.76 (1.45–5.28)**	**<0.01**	**3.22 (1.65–6.27)**	**<0.01**
15	32 (31.1%)	71 (68.9%)	**2.70 (1.36–5.38)**	**<0.01**	**3.39 (1.67–6.90)**	**<0.01**
24	44 (28.9%)	108 (71.1%)	**2.44 (1.28–4.68)**	**<0.01**	**2.90 (1.48–5.66)**	**<0.01**
25	34 (29.8%)	80 (70.2%)	**2.55 (1.29–5.02)**	**<0.01**	**3.10 (1.54–6.23)**	**<0.01**
34	19 (13.1%)	126 (86.9%)	0.91 (0.44–1.88)	0.79	1.03 (0.49–2.16)	0.94
35	13 (12.3%)	93 (87.7%)	0.84 (0.38–1.86)	0.66	0.89 (0.40–2.01)	0.78
44	26 (18.2%)	117 (81.8%)	1.33 (0.67–2.66)	0.41	1.49 (0.73–3.03)	0.27
45	15 (14.3%)	90 (85.7%)	1		1	
AGE OF THE CHILD AT THE TIME OF TREATMENT (YEARS)						
≤4	6 (7.6%)	16 (11.8%)	1		–	–
5	15 (19.0%)	24 (17.6%)	1.49 (0.48–4.69)	0.49	–	–
6	18 (22.8%)	37 (27.2%)	1.30 (0.43–3.88)	0.64	–	–
7	40 (50.6%)	59 (43.4%)	1.81 (0.65–5.02)	0.26	–	–

## Discussion

The evaluation of potential adverse effects of drugs used in pulp therapy on primary molars on the enamel of successor premolars in formation is important to prevent irreversible alterations.^9,16,19^ This is the first study that, according to the criteria recommended by the Federation Dental International (1992),^12^ which evaluates DDE in premolars whose predecessors with pulp necrosis were treated with antibiotic paste.

CTZ paste presents as a differential compared to conventional endodontic techniques, the ease of execution, since it does not require the chemical-mechanical preparation phase of root canals, in addition to its low cost.^2,4,6,7,11^ This simplified technique allows for execution by general clinicians who treat children and thus prevents early extraction of primary molars and consequent malocclusions.^11,20,26^

The permeability of the pulpal chamber floor of primary molars allows for communication between the pulp chamber and the periodontal region, favouring the dissemination of inflammatory mediators, bacterial metabolism products and/or drugs present in endodontic materials that can trigger DDE in premolars.^13,16,19^ The literature lacks evidence regarding the action of antibiotic pastes for endodontic use in primary teeth on periradicular tissues and permanent successors.^14^

When used during amelogenesis, CTZ paste may represent a risk factor for colour changes and/or enamel hypoplasia in permanent successor teeth due to the presence of tetracycline in its composition.^23^ In primary molars treated with CTZ paste, the darkening of the tooth structure is clinically observed. However, there is no evidence regarding the topical use of antibiotics in primary molars and their action on successive premolars, but it is known that the chemical structure of tetracycline presents sites with a high affinity for calcium ions, capable of establishing stable bonds and causes colour changes when in contact with the mineralised tissues.^23^

A higher frequency of DDE in premolars whose predecessors presented with pulp necrosis was observed. This result provides evidence that pulp necrosis in primary molars, regardless of the treatment adopted, represents a risk factor for defects in the enamel of premolars. In addition, there is a greater chance of premolars having DDE when their predecessors were extracted due to caries and consequent pulp necrosis. Although follicles of permanent teeth have a defence mechanism against pulpal infections in predecessors, with the formation of fibrous tissue surrounding them,^9,18^ persistent infectious processes that can cause irreversible damage to the enamel of the developing successor permanent teeth.^9^

The frequency of DDE was significantly lower in premolars whose predecessors were treated with CTZ paste when compared to the group in which an extraction was performed due to untreated necrosis. These data may be related to the interruption of the infectious process caused by the action of antibiotics, evidenced by the good clinical and radiographic results of the CTZ paste.^11^

Demarcated opacity was the most frequent defect, followed by hypoplasia. Clinically, both defects are limited and restricted to a few teeth and, in general, have been attributed to local causes such as infections and dentoalveolar trauma.^5^ Hypoplastic defects are caused by disturbances in the secretory stage of amelogenesis, in which ameloblasts secrete an organic matrix that determines the enamel thickness.^21,24^ Clinically, teeth with hypomineralisation present opacities, which are the result of injuries in the maturation phase related to the degradation of the organic matrix and the growth of hydroxyapatite crystals.^21,24^ The higher occurrence of demarcated opacities is justified by the prolonged duration of the maturation stage when compared to that of secretion.^25^

Maxillary premolars presented with a higher frequency of DDE when compared to mandibular premolars. The lower alveolar bone density of the maxilla may also favour greater exposure of the developing permanent tooth to periradicular/inflammation infection products of primary teeth.^1^ Additionally, accessory ducts are more numerous in maxillary primary molars.^16^ This fact could contribute to the fact that their predecessors showed a greater permeability in the pulp chamber floor. However, there is little evidence of anatomical descriptions of the furcation region and its association with the permeability of primary molars.^13,16,19^ The age of the child at the time of extraction or pulp therapy was not associated with the presence of defects, although it has been reported that the earlier stages of dental development present a greater predisposition to amelogenesis disorders when exposed to risk factors.^25^ However, the results should be interpreted with caution because it is impossible to determine the exact time when inflammatory and infectious processes were initiated and that permanent teeth which were forming remained exposed to inflammatory mediators and/or toxins from pulp necrosis until treatment. Prospective studies using more robust methodologies are needed, given the limitations of the observational studies, which are important for hypothesis elaboration.

Possible limitations regarding data collection in dental records have been overcome, since in the records of the dental clinic, consent and signature of planning and treatment by the responsible teacher were required. Also, in the group treated with CTZ paste no information was evaluated about the clinical and radiographic outcome of the pulp therapy. The risk of blinding-related bias was minimised because the examiner was unaware of which group the premolars examined belonged to.

## Conclusion

The prevalence of enamel defects was higher in premolars whose predecessors with pulp necrosis were extracted, followed by those with pulp necrosis treated with CTZ paste and healthy teeth.
